# Trigeminal-Rostral Ventromedial Medulla circuitry is involved in orofacial hyperalgesia contralateral to tissue injury

**DOI:** 10.1186/1744-8069-8-78

**Published:** 2012-10-23

**Authors:** Bryan Chai, Wei Guo, Feng Wei, Ronald Dubner, Ke Ren

**Affiliations:** 1 Department of Neural and Pain Sciences, School of Dentistry, University of Maryland, Baltimore, MD, 21201, USA; 2 Program in Neuroscience, University of Maryland, Baltimore, MD, 21201, USA

**Keywords:** Pain, Descending modulation, Serotonin, Substance P, IL-1β, Vi/Vc transition zone, Rat

## Abstract

**Background:**

Our previous studies have shown that complete Freund’s adjuvant (CFA)-induced masseter inflammation and microinjection of the pro-inflammatory cytokine interleukin-1β (IL-1β) into the subnucleus interpolaris/subnucleus caudalis transition zone of the spinal trigeminal nucleus (Vi/Vc) can induce contralateral orofacial hyperalgesia in rat models. We have also shown that contralateral hyperalgesia is attenuated with a lesion of the rostral ventromedial medulla (RVM), a critical site of descending pain modulation. Here we investigated the involvement of the RVM-Vi/Vc circuitry in mediating contralateral orofacial hyperalgesia after an injection of CFA into the masseter muscle.

**Results:**

Microinjection of the IL-1 receptor antagonist (5 nmol, n=6) into the ipsilateral Vi/Vc attenuated the CFA-induced contralateral hyperalgesia but not the ipsilateral hyperalgesia. Intra-RVM post-treatment injection of the NK1 receptor antagonists, RP67580 (0.5-11.4 nmol) and L-733,060 (0.5-11.4 nmol), attenuated CFA-induced bilateral hyperalgesia and IL-1β induced bilateral hyperalgesia. Serotonin depletion in RVM neurons prior to intra-masseter CFA injection prevented the development of contralateral hyperalgesia 1–3 days after CFA injection. Inhibition of 5-HT_3_ receptors in the contralateral Vi/Vc with direct microinjection of the select 5-HT_3_ receptor antagonist, Y-25130 (2.6-12.9 nmol), attenuated CFA-induced contralateral hyperalgesia. Lesions to the ipsilateral Vc prevented the development of ipsilateral hyperalgesia but did not prevent the development of contralateral hyperalgesia.

**Conclusions:**

These results suggest that the development of CFA-induced contralateral orofacial hyperalgesia is mediated through descending facilitatory mechanisms of the RVM-Vi/Vc circuitry.

## Background

Orofacial muscle pain is a debilitating disorder that diminishes the quality of life and seriously inhibits the health of the patient by impairing a person’s ability to eat and drink. Trigeminal pain also spreads to wide orofacial areas and is a serious clinical problem. Reports have shown that patients with unilateral myofascial temporomandibular muscle and joint disorders (TMD) experience bilateral hyperalgesia [1,2]. Very little research has focused on the possible circuitry and mechanisms involved in the development of contralateral orofacial hyperalgesia after deep tissue orofacial injury.

Traditionally, orofacial pain research has focused on the activation of the subnucleus caudalis (Vc) of the spinal trigeminal nucleus (STN) [3-7]. Despite the emphasis of orofacial pain research in the Vc, the subnucleus interpolaris/subnucleus caudalis transition zone (Vi/Vc) of the STN has been more recently shown to be involved in mechanisms of deep tissue trigeminal pain [8-11]. We have shown that complete Freund’s adjuvant (CFA)-induced masseter inflammation and intra-Vi/Vc microinjection of the pro-inflammatory cytokine, interleukin-1β (IL-1β), induce contralateral orofacial hyperalgesia in rat [12,13]. However, contralateral hyperalgesia is not seen with intra-Vc IL-1β microinjection. Similar to the behavioral findings, our previous studies showed that CFA-induced masseter inflammation results in bilateral expression of Fos protein in the Vi/Vc and ipsilateral expression of Fos protein in the Vc [14].

We have also shown that IL-1β-induced contralateral hyperalgesia is attenuated with ibotenic acid lesions in the rostral ventromedial medulla (RVM), a midline site that is critical in descending pain modulation [12]. Our previous studies have shown that bilateral reciprocal connections exist from the Vi/Vc to the RVM [14]. This suggests that activation of RVM neurons may be necessary for the development of contralateral hyperalgesia. Immunohistochemical localization shows moderate amounts of the substance P receptor, neurokinin-1 receptor (NK1-R), in the RVM [15-17]. Research has shown that Substance P activation of NK1-R in the RVM is involved in descending facilitation of pain [18-25]. We have also shown that the serotonin system is involved in descending facilitation in the spinal cord after tissue and nerve injury [26]. Furthermore, spinal 5-HT_3_ receptor activation has been shown to contribute to the development of hyperalgesia after spinal cord injury [27,28].

Based on these studies of descending facilitation and behavioral hyperalgesia at the spinal and trigeminal levels, we propose that the activation of the NK1-R in the RVM by input from the ipsilateral Vi/Vc results in enhanced release of 5-HT from descending RVM-Vi/Vc neurons. Activation of 5-HT_3_ receptors in the contralateral Vi/Vc ultimately leads to contralateral hyperalgesia.

## Results

### Contralateral orofacial hyperalgesia is mediated via IL-1R activation in the ipsilateral Vi/Vc

**Figure 1:**
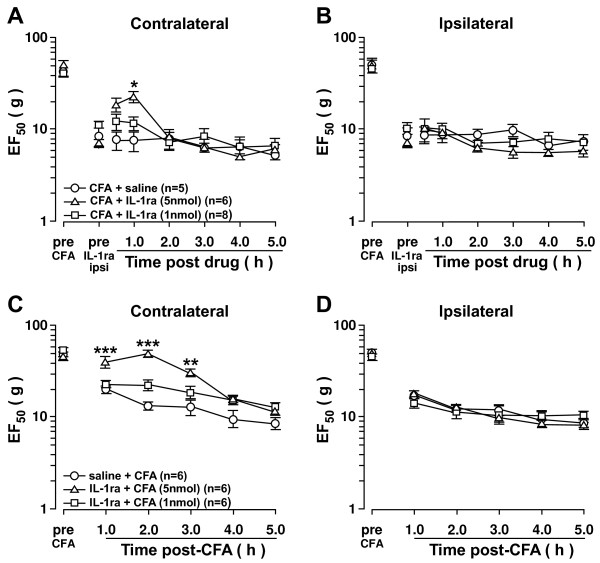
**Inhibition of IL-1R in the ipsilateral Vi/Vc attenuates contralateral hyperalgesia.** Contralateral orofacial hyperalgesia is attenuated by IL-1R inhibition in the ipsilateral Vi/Vc. **A**, **B**. Post-treatment: CFA (0.05 ml; 1:1 oil/saline) was injected unilaterally into the masseter muscle 24 h prior to IL-1ra microinjection into the ipsilateral Vi/Vc (0.5μl). **C**, **D**. Pre-treatment: CFA (0.05 ml; 1:1 oil/saline) was injected unilaterally into the masseter muscle 15 min after IL-1ra microinjection into the ipsilateral Vi/Vc (0.5 μl). Pre-treatment and post-treatment of IL-1ra attenuated CFA-induced contralateral hyperalgesia as compared to saline treated rats. IL-1ra did not attenuate ipsilateral hyperalgesia. *: IL-1ra (5 nmol) vs saline. *: p<;0.05; **: p<;0.01; ***: p<;0.001 (ANOVA with repeated measures and Student Neuman-Keuls post-hoc test).

Our previous studies have shown that CFA injection into the masseter muscle and IL-1β injections into the Vi/Vc transition zone generate bilateral mechanical hyperalgesia as seen by the significant decreases in EF_50_ values [12,13]. To test whether activation of IL-1 receptors (IL-1R) in the Vi/Vc is involved in CFA-induced contralateral hyperalgesia, an IL-1R antagonist (IL-1ra) was injected. Microinjection of IL-1ra (5 nmol, n=6) into the ipsilateral Vi/Vc 24 h after CFA treatment attenuated contralateral hyperalgesia 1 h after microinjection as compared to saline-treated rats (Figure 1A). Intra-Vi/Vc pretreatment of IL-1ra (5 nmol, n=6) also attenuated CFA-induced contralateral hyperalgesia compared to saline-treated rats 1 h after CFA injection (Figure 1C). Pre-treatment and post-treatment of IL-1ra did not attenuate ipsilateral hyperalgesia (Figure 1B, D). These results suggest that IL-1R in the ipsilateral Vi/Vc is involved in CFA-induced contralateral orofacial hyperalgesia but not the ipsilateral orofacial hyperalgesia.

### NK1-R activation in the RVM is critical to the development of bilateral orofacial hyperalgesia

**Figure 2:**
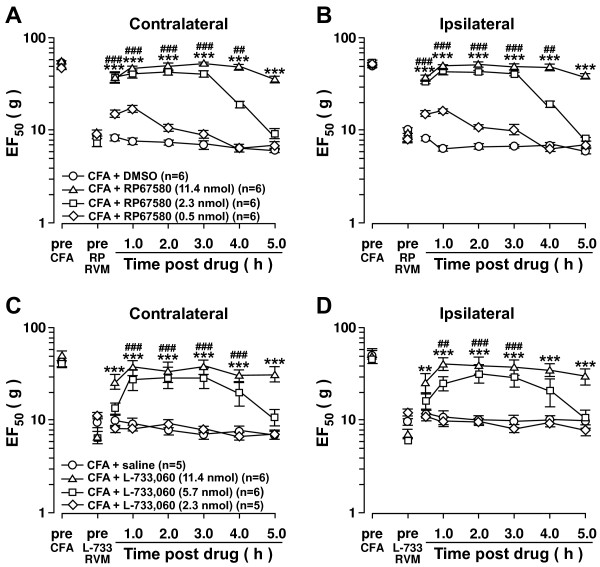
**Inhibition of NK1-R in the RVM attenuates inflammatory bilateral hyperalgesia.** Intra-RVM microinjection of NK1-R antagonists attenuates CFA-induced bilateral hyperalgesia. CFA (0.05 ml; 1:1 oil/saline) was injected unilaterally into the masseter muscle 24 h prior to RP67580 (**A**, **B**) and L-733,060 (**C**, **D**) microinjection into the RVM (0.5 μl). +: lowest dose vs. saline. #: middle dose vs. saline. *: highest dose vs. saline. +,#,*: p<;0.05; ++,##,**: p<;0.01; +++,###,***: p<;0.001 (ANOVA with repeated measures and Student Neuman-Keuls post-hoc test).

Previously we showed that intra-Vi/Vc IL-1β-induced contralateral orofacial hyperalgesia was mediated via RVM neurons [12]. The RVM is a midline structure that lacks laterality and projects neurons bilaterally to the ventral Vi/Vc [14]. NK1-R activation in the RVM has been shown to contribute to capsaicin-induced hyperalgesia [22]. Similarly, we showed that NK1-R activation in the RVM is involved in mediating behavioral hyperalgesia after hindpaw inflammation [24]. To test whether NK1-R activation in the RVM was involved in orofacial contralateral hyperalgesia, two NK1-R antagonists, RP67580 and L-733,060 were microinjected into the RVM. RP67580 attenuated CFA-induced bilateral hyperalgesia in a dose-dependent manner. Attenuation of hyperalgesia was seen 0.5-5 h post-injection with the highest dose of RP67580 (11.4 nmol; n=6) on the contralateral side (Figure 2A) and ipsilateral side (Figure 2B). Similar results were observed using the NK1-R antagonist L-733,060 (Figure 2C, D).

**Figure 3:**
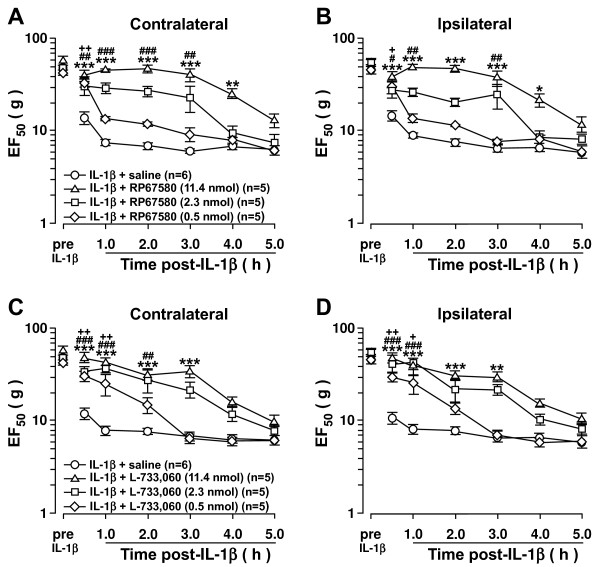
**Inhibition of NK1-R in the RVM attenuates IL-1β-induced bilateral hyperalgesia.** Intra-RVM microinjection of NK1-R antagonists attenuates intra-Vi/Vc IL-1β-induced bilateral hyperalgesia. IL-1β (160 fmol/0.5 μl) was microinjected unilaterally into the Vi/Vc 15 min prior to RP67580 (**A**, **B**) and L-733,060 (**C**, **D**) microinjection into the RVM (0.5 μl). +: lowest dose vs. saline. #: middle dose vs. saline. *: highest dose vs. saline. +,#,*: p<;0.05; ++,##,**: p<;0.01; +++,###,***: p<;0.001 (ANOVA with repeated measures and Student Neuman-Keuls post-hoc test).

Inhibition of NK1-R activation in the RVM also attenuated IL-1β (160 fmol) – induced bilateral orofacial hyperalgesia. Microinjection of RP67580 and L-733,060 into the RVM 15 min after IL-1β injection into the Vi/Vc resulted in a dose-dependent attenuation of bilateral hyperalgesia. Application of RP67580 (11.4 nmol; n=5) resulted in attenuation of hyperalgesia 0.5-4 h post-injection on the contralateral side (Figure 3A) and ipsilateral side (Figure 3B). Similar results were observed using NK1-R antagonist L-733,060 (Figure 3C, D). These results suggest that contralateral and ipsilateral orofacial hyperalgesia are mediated by NK1-R activation in the RVM.

### 5-HT depletion in the RVM inhibits the development of CFA-induced contralateral orofacial hyperalgesia

**Figure 4:**
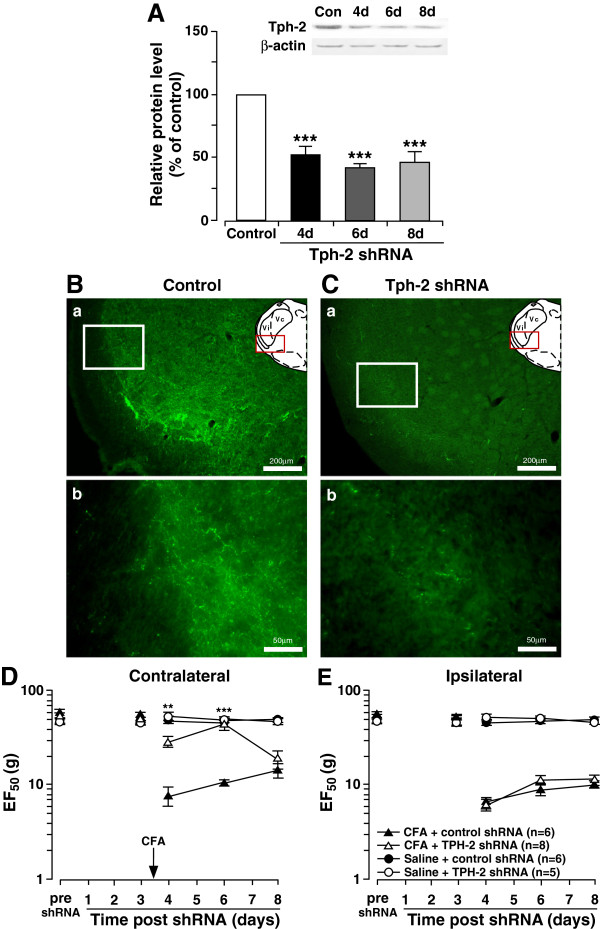
**5-HT depletion in the RVM prevents the development of contralateral hyperalgesia.** RNAi of Tph-2 in the RVM prevents the development of CFA-induced contralateral hyperalgesia. **A**. Western blot analysis of Tph-2 in the RVM. CFA (0.05 ml; 1:1 oil/saline) injected unilaterally into the masseter muscle 3 days after shRNA administration. Tph-2 protein expression was measured in rats at 4 day (n=4), 6 days (n=4) and 8 days (n=4) after shRNA was administered. Tph-2 protein expression was measured in rats given control shRNA (n=4) 4 days after the shRNA was administered. **B**, **C**. Immunohistochemical fluorescent staining of 5-HT in the Vi/Vc of shRNA treated rats (n=3) 24 h after CFA administration shows decreased 5-HT expression in the Vi/Vc following Tph-2 depletion in rats as compared to control shRNA (n=3). CFA was administered 3 days after shRNA administration. (Ba: control shRNA, 10x; Bb: control shRNA,40x. Ca: Tph-2 shRNA,10x; Cb: Tph-2 shRNA,40x). **D**, **E**. Mechanical hyperalgesia was tested in 5-HT depleted rats with CFA-induced hyperalgesia. Control or Tph-2 shRNA was administered and CFA was injected 3 days after. Mechanical hyperalgesia was inhibited in 5-HT depleted rats as compared to control on the contralateral side (**D**), but not the ipsilateral side (**E**). *: CFA + Tph-2 shRNA vs. CFA + control shRNA, p<;0.05; **: p<;0.01; ***: p<;0.001 (ANOVA with repeated measures and Student Neuman-Keuls post-hoc test).

The results suggest that NK1-R activation of RVM neurons may lead to descending facilitation of pain on the contralateral side and ipsilateral side. We previously showed that depletion of 5-HT in the RVM neurons inhibits injury-induced hyperalgesia [26] and an intrathecal 5-HT_3_ receptor antagonist blocked intra-RVM SP-induced hyperalgesia [24]. Thus, we tested the hypothesis that NK1-R activation in the RVM could ultimately result in 5-HT-dependent descending facilitation to the contralateral Vi/Vc. To evaluate the role of 5-HT in the development of contralateral orofacial hyperalgesia, down regulation of tryptophan hydroxylase-2 (Tph-2), a rate limiting enzyme for 5-HT synthesis in RVM neurons was achieved by RNA interference. Since the RVM is a midline structure that projects bilaterally, RNA interference would result in bilateral 5-HT depletion at the Vi/Vc. In CFA-induced hyperalgesic rats, western blot showed that Tph-2 expression in the RVM was significantly down regulated at 4–8 days after Tph-2 shRNA administration when compared to control (n=4; p<;0.001) (Figure 4A). Immunostaining at 4 days after shRNA administration shows a reduction of 5-HT immunoreactivity in the ventral Vi/Vc axons of CFA-induced hyperalgesic rats when compared to control shRNA (Figure 4B, C). 5-HT depletion inhibits the development of CFA-induced contralateral orofacial hyperalgesia 4–6 days after CFA injection (Figure 4D). However, 5-HT depletion did not inhibit development of ipsilateral hyperalgesia after CFA masseter muscle injection (Figure 4E). These data suggests that 5-HT-dependent descending facilitation of pain is necessary for the development of contralateral hyperalgesia but not the ipsilateral hyperalgesia.

### Vi/Vc 5-HT_3_ receptor activation is necessary for CFA-induced contralateral orofacial hyperalgesia

**Figure 5:**
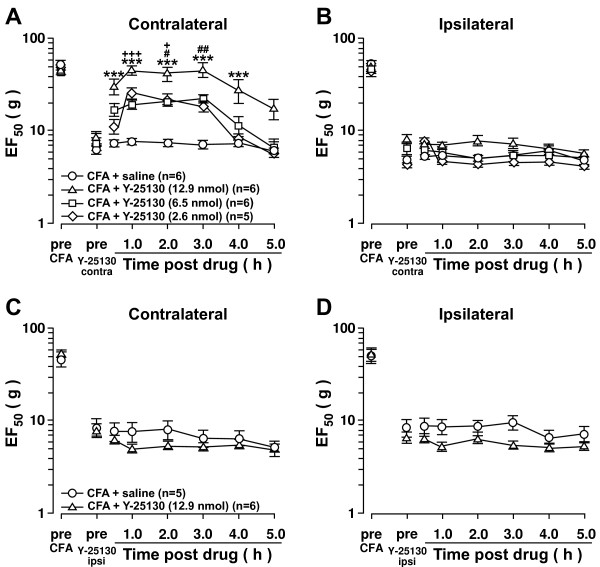
**Vi/Vc 5-HT**_**3**_**receptor inhibition attenuates contralateral hyperalgesia.** Inhibition of the 5-HT_3_ receptor in the contralateral Vi/Vc attenuates CFA-induced contralateral hyperalgesia. CFA (0.05 ml; 1:1 oil/saline) was injected into the masseter muscle and 24 h later, Y-25130 was microinjected (0.5 μl) into the contralateral Vi/Vc (**A**, **B**). Y-25130 microinjection into the contralateral Vi/Vc attenuated contralateral hyperalgesia (**A**) but not ipsilateral hyperalgesia (**B**). Y-25130 microinjection into the ipsilateral Vi/Vc (**C**, **D**) did not attenuate ipsilateral or contralateral hyperalgesia. +: lowest dose vs. saline. #: middle dose vs. saline. *: highest dose vs. saline. +,#,*: p<;0.05; ++,##,**: p<;0.01; +++,###,***: p<;0.001 (ANOVA with repeated measures and Student Neuman-Keuls post-hoc test).

Previous studies have shown that 5-HT_3_ receptor expression is upregulated in the spinal cord and trigeminal nuclei in animal models of persistent pain [29,30]. In similar models of persistent pain, hyperalgesia is attenuated following 5-HT_3_ receptor inhibition [28,29,31]. To test whether the 5-HT_3_ receptor was involved in contralateral orofacial hyperalgesia, a 5-HT_3_ receptor antagonist, Y-25130 was microinjected into the contralateral Vi/Vc following CFA masseter muscle injection. Contralateral orofacial hyperalgesia was dose-dependently attenuated following Y-25130 (n=6) (Figure 5A). In contrast, Y-25130 microinjected into the contralateral Vi/Vc did not attenuate the ipsilateral hyperalgesia (Figure 5B). Microinjection of Y-25130 into the ipsilateral Vi/Vc did not attenuate either ipsilateral or contralateral hyperalgesia (Figure 5C, D). These results suggest that 5-HT_3_ receptor activation in the contralateral Vi/Vc facilitates the CFA-induced contralateral hyperalgesia but may not be necessary for the CFA induced ipsilateral hyperalgesia.

### Ipsilateral Vc input is necessary for the development of CFA-induced ipsilateral orofacial hyperalgesia

**Figure 6:**
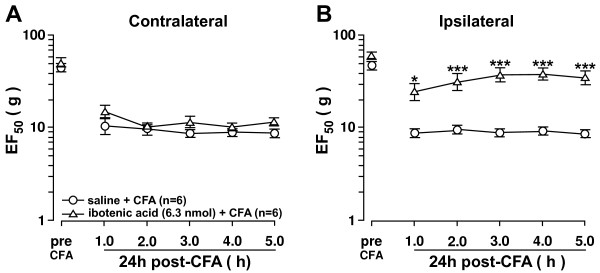
**Vc activation is necessary for the development of ipsilateral hyperalgesia.** Lesion of the ipsilateral Vc prior to CFA injection into the master muscle prevents the development of ipsilateral hyperalgesia. Ibotenic Acid (6.3 nmol) was administered to unilaterally to the Vc between vertebrae C1-C2, 7 days prior to ipsilateral CFA injection into the masseter muscle. Lesion of the ipsilateral Vc did not prevent the development of contralateral hyperalgesia (**A**). However, lesion of the ipsilateral Vc did prevent the development of ipsilateral hyperalgesia (**B**). *: Ibotenic Acid (6.3 nmol) vs. saline, p<;0.05; **: p<;0.01; ***: p<;0.001 (ANOVA with repeated measures and Student Neuman-Keuls post-hoc test).

The present data provide evidence that Vi/Vc was necessary for contralateral orofacial hyperalgesia. Our present data also showed that injection of IL-1ra into the Vi/Vc did not affect the ipsilateral hyperalgesia. Our previous studies have shown that afferent nociceptive input into the Vc is critical for the development of ipsilateral hyperalgesia but does not affect contralateral hyperalgesia [13]. This suggests that the ipsilateral and contralateral hyperalgesia may be transmitted through different circuits in the trigeminal system. To test this, a lesion was made in the ipsilateral Vc using 6.3 nmol of the excitotoxin, ibotenic acid. Ibotenic acid induces glutamate-mediated toxicity to neuron cell bodies through activation of glutamate receptors. An ipsilateral Vc lesion prior to CFA injection inhibited the development of ipsilateral hyperalgesia (Figure 6B) but did not inhibit contralateral hyperalgesia development (Figure 6A). This suggests that ipsilateral Vc input is primarily involved in the development of ipsilateral hyperalgesia while Vi/Vc input is necessary for the development of hyperalgesia contralateral to the site of injury.

### Histological confirmation of injection sites in the RVM and Vi/Vc transition zone

**Figure 7:**
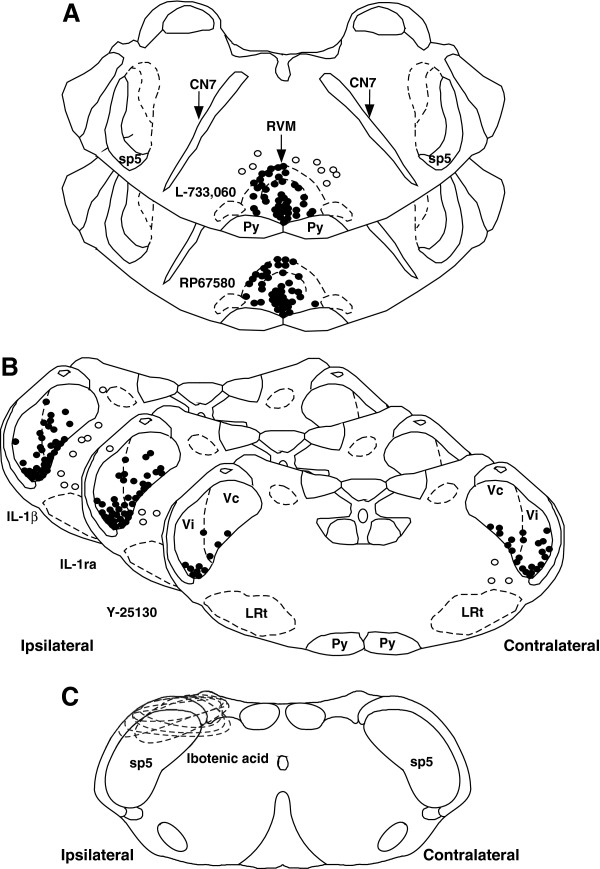
**Histological representation of injection sites within the RVM and Vi/Vc.** Histological representation of the cannula tip placement within the RVM (**A**), the Vi/Vc transition zone (**B**), and Vc (**C**). Diagrams are separated according to the drug administered. RVM: L-733,060 and RP67580; Vi/Vc: IL-1β. IL-1ra, and Y-25130; Vc: ibotenic acid. Closed circles represent cannula tips accurately placed within the site of interest. Open circles represent cannula tips placed outside the site of interest. Dashed circles represent affected area from ibotenic acid injection, which involved dorsal mandibular and middle maxillary divisions. Animals with missed sites of injection showed no significant effect compared to control and were excluded from experimental groups. CN 7: facial nerve; sp5: Spinal trigeminal nucleus; Py; pyramidal tract; LRt: lateral reticulate nucleus.

Animals were perfused with 4% paraformaldehyde at the conclusion of the experiment and the sections of brain stem tissues were stained with cresyl violet for histological verification of the sites of injection in the Vi/Vc, RVM, and Vc (Figure 7A, B, C). Animals with missed sites of injection showed no significant effect compared to control and were excluded from experimental groups.

## Discussion

Our previous studies have shown that CFA-induced masseter inflammation and intra-Vi/Vc microinjection of IL-1β result in contralateral orofacial hyperalgesia [12,13]. We have also shown that lesions in the RVM prevented the development of contralateral hyperalgesia [12]. The present study further shows that the development of contralateral orofacial hyperalgesia involves IL-1R activation in the ipsilateral Vi/Vc that ultimately leads to NK1-R activation in the RVM and 5-HT_3_ receptor activation in the contralateral Vi/Vc.

The RVM is a critical site in descending modulation of pain [32,33]. Much research has focused on the descending inhibitory affects of the opioid system within the RVM [34,35]. Our lab has shown that upregulation and phosphorylation of AMPA receptors in the RVM can lead to descending inhibition of inflammatory pain [36,37]. However, there has been mounting evidence of RVM-mediated descending facilitation [38-42]. The RVM has been shown to have reciprocal connections with trigeminal neurons in the Vi/Vc transition zone [14]. Our previous studies indicated that intra-masseter muscle CFA injection and intra-Vi/Vc IL-1β injection produces bilateral orofacial hyperalgesia. The RVM was previously shown to mediate the IL-1β-induced hyperalgesia on the contralateral side [12].

The contralateral effect was also produced by an IL-1β injection into the Vi/Vc but was not produced after IL-1β injection into the Vc [13]. Rats with masseter muscle inflammation exhibit increased IL-1β expression in Vi/Vc astroglia [43]. Furthermore, IL-1 receptor (IL-1R) activation induces a protein kinase-C dependent signaling cascade that leads to N-methyl-D-aspartate receptor phosphorylation and ultimately behavioral hyperalgesia [43]. In our present study, inhibition of IL-1R activation in the ipsilateral Vi/Vc before CFA treatment prevented the development of contralateral orofacial hyperalgesia. Similarly, inhibition of IL-1R activation in the ipsilateral Vi/Vc after CFA treatment resulted in an attenuation of the contralateral hyperalgesia. These results support the view that activation of IL-1R in the ipsilateral Vi/Vc is involved in the development and maintenance of deep tissue orofacial pain on the contralateral site.

Substance P has been shown to be involved in RVM descending modulation of pain [22,25]. NK1-R activation in the RVM enhances excitatory glutamatergic inputs to RVM neurons in rats with persistent pain [44]. NK1-R antagonism in the RVM attenuated CFA-induced thermal hyperalgesia in the hind paw [23]. Our previous studies have shown that NK1-R expression is increased in the RVM following hind paw inflammation and direct injection of substance P into the RVM can induce bilateral thermal hyperalgesia in the hind paw [24]. We also showed that Substance P-induced hyperalgesia can be mediated by 5-HT, NMDA, and GABA_A_ circuits [24]. We have now shown that NK1-R inhibition in the RVM attenuated inflammatory orofacial hyperalgesia bilaterally. These results suggest that NK1-R activation in the RVM contributes to descending facilitation that mediates inflammatory orofacial hyperalgesia to the ipsilateral and contralateral side.

In the spinal cord, administration of a 5-HT_3_ receptor antagonist inhibits evoked responses of dorsal horn neurons after noxious stimulation [29] and intra-RVM cholecystokinin-induced mechanical and thermal hyperalgesia [45]. Similarly, intrathecal administration of a 5-HT_3_ receptor antagonist attenuates formalin-induced hind paw flinching and spinal ERK activation [46]. We have previously shown that after the depletion of endogenous 5-HT while maintaining the viability and function of serotonergic neurons, descending facilitation is dependent on the activation of serotonergic neurons in the RVM [26]. The present study shows that descending serotonergic facilitation is necessary for the development and maintenance of contralateral orofacial hyperalgesia after CFA-induced masseter inflammation. Furthermore, activation of Vi/Vc 5-HT_3_ receptors is necessary for induction of the CFA-induced contralateral orofacial hyperalgesia to develop.

Interestingly, our results show that IL-1R inhibition and 5-HT_3_ receptor blockade in the ipsilateral Vi/Vc does not affect ipsilateral hyperalgesia. This finding suggests that Vc activation from afferent input is important for the ipsilateral hyperalgesia while the development of contralateral hyperalgesia requires Vi/Vc-RVM circuitry. Supporting this hypothesis, lesions of the Vc inhibited the development of ipsilateral hyperalgesia without affecting contralateral hyperalgesia. It appears that the afferent drive into the Vc is the primary mechanism involved in ipsilateral hyperalgesia consistent with our previous findings [13].

A puzzling observation from this study is that shRNA treatment of Tph-2 in the RVM, which down regulates 5-HT bilaterally, did not affect ipsilateral hyperalgesia. One would expect that hyperalgesia would be attenuated bilaterally. One possible explanation is that ipsilateral hyperalgesia is dominated by hyperexcitability and central sensitization in the Vc driven by peripheral input which masks the loss of descending facilitation. An alternative explanation is that the absence of attenuation of ipsilateral hyperalgesia may be related to the finding of varying degrees of descending inhibitory and facilitatory input into the contralateral and ipsilateral medullary/spinal dorsal horn. It has been shown that descending pain modulation is in dynamic balance between inhibitory and facilitatory signals [47]. After hind paw inflammation, there is a time-dependent enhancement of descending inhibition on the ipsilateral side [48,49]. Descending 5-HT projections from the RVM also produce dual inhibitory and facilitatory effects due to activation of different 5-HT receptor subtypes [50]. The net effect of descending modulation may be facilitatory on the contralateral side and inhibitory on the ipsilateral side in this masseter inflammation model. Thus, depletion of descending 5-HT on the ipsilateral side would reduce predominant inhibitory signals and the hyperalgesia would be sustained.

## Conclusions

**Figure 8:**
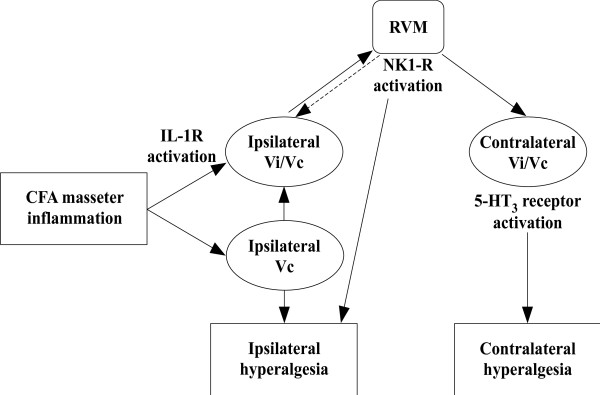
**Vi/Vc-RVM circuitry is necessary for the development of contralateral hyperalgesia.** The proposed pathway involved in the development of contralateral orofacial hyperalgesia following unilateral deep tissue inflammatory hyperalgesia. Unilateral CFA masseter inflammation leads to increased IL-1β activation of IL-1R in the ipsilateral Vi/Vc. Direct or indirect activation of the RVM through NK1-R activation leads to descending serotonergic facilitation of pain to the contralateral Vi/Vc. Activation of 5-HT_3_ receptors in the contralateral Vi/Vc leads to contralateral orofacial hyperalgesia.

Overall, our results support the hypothesis that the Vi/Vc–RVM circuitry mediates the development of hyperalgesia on the contralateral side after ipsilateral deep tissue injury. CFA-induced inflammatory orofacial hyperalgesia produces an increase in IL-1β activation of IL-1R in the ipsilateral Vi/Vc. IL-1R activation in the Vi/Vc ultimately leads to NK1-R activation in the RVM. This leads to activation of descending serotonin-containing RVM neurons that project to the Vi/Vc bilaterally. Activation of 5-HT_3_ receptors in the contralateral Vi/Vc results in deep tissue orofacial pain in the contralateral masseter muscle (Figure 8). The findings further suggest that descending facilitatory mechanisms may also contribute to the development of referred pain at widespread sites via RVM descending circuitry.

## Methods

### Animals

Male Sprague–Dawley rats (n=222 total) weighing 200-300g (Harlan) were used for all experiments. Female rats were excluded to avoid complications of the estrous cycle. Rats were provided food and water *ad libitum* on a 12-h light/dark schedule. The experiments were approved by the Institutional Animal Care and Use Committee at the University of Maryland Dental School.

### Surgical preparation/cannulation

Rats were anesthetized using 50 mg/kg of pentobarbital sodium (i.p) and 2-3% isoflurane inhalation in a 30/70% oxygen/nitrogen gas mixture. Rats were placed in a stereotaxic device (Kopf Instruments, Model 900). A midline incision was made in the scalp after debridement and sterilization of the surgical field with iodine wash. For administration of drugs via microinjection, guide cannulae (C315G, 26 gauge, Plastics One, Roanoke, VA) were implanted and cemented into the skull. A midline opening was made in the skull using a dental drill and a guide cannula was lowered into the ventral Vi/Vc transition zone or RVM by referring to the rat brain atlas [51]. The RVM is termed for the collective structures that consist of the midline nucleus raphe magnus (NRM) and the adjacent gigantocellular reticular nucleus pars α (NGC α). The guide cannula was then secured with cranioplastic cement. The wounds were cleaned with antiseptic solution and closed with 4–0 silk sutures. Animals were allowed to recover for 1 week before further experimentation. A group of rats received unilateral intra-masseter muscle injections of the inflammatory agent, complete Freund’s adjuvant (CFA, 0.05ml; 1:1 oil/saline). Twenty-four hours later, drugs were injected into the ventral Vi/Vc transition zone or RVM through a 33-gauge injection cannula (C315I, Plastic One) inserted through the tip of the guide cannula. The injection cannula was connected to a 1-μl Hamilton syringe by polyethylene-10 tubing. All injections (0.5 μl) were performed by delivering drug or vehicle solutions slowly over a 2-min period. Recombinant rat IL-1β, IL-1 receptor antagonist (PeproTech; Rocky Hill, NJ) and 5-HT_3_ receptor antagonist Y-25130 (Tocris Bioscience; Ellisville, MO) were microinjected into the Vi/Vc transition zone. The Neurokinin-1 receptor antagonists L-733,060 and RP67580 (Tocris Bioscience; Ellisville, MO) were microinjected into the RVM. IL-1β, IL-1 receptor antagonist, Y-25130, and L-733,060 were reconstituted in deionized water while RP67580 was reconstituted in DMSO.

### shRNA

shRNA plasmids containing the 5-HT synthesizing enzyme Tryptophan Hydroxylase – 2 (Tph-2) or a scrambled sequence control was administered into the RVM with a 1-μL Hamilton syringe (0.5 μg/0.5 μl) over 5 min. Fifteen minutes after the injection, the syringe was slowly removed and a pair of Teflon-coated silver positive and negative electrodes spaced 3 mm apart were placed in a rostrocaudal direction at the injection site for electroporation (7 square-wave pulses; 50 ms, 40 V, 1 Hz). The rats were allowed to heal for 3 days before unilateral masseter injection of CFA. Behavioral tests were performed 24 h post-CFA.

### Western blot

Naïve and treated rats were anesthetized with 50mg/kg pentobarbital sodium (i.p) and decapitated. The RVM tissue was removed as previously described [44]. The RVM tissue was homogenized and centrifuged at 20,200 x g for 10 min at 4°C, and the supernatant was removed. The protein concentration was determined. Each sample contained proteins from one animal. The proteins (50 μg) were separated on a 7.5% SDS-PAGE gel and blotted to a nitrocellulose membrane (GE Healthcare Biosciences, Pittsburgh, PA). The blot was incubated with rabbit anti-Tph-2 antibody overnight at 4°C. The membrane was washed with TBS and incubated for 1 h with anti-goat IgG horseradish peroxidase (HRP) (1:3000; Santa Cruz Biotechnology, Santa Cruz, CA) in 5% milk/TBS. The immunoreactivity was detected using enhanced chemiluminescence (ECL) (GE Healthcare, Pittsburgh, PA). The loading and blotting of equal amount of proteins were verified by reprobing the membrane with anti-β-actin antiserum (Sigma, St. Louis, MO). The ECL-exposed films were digitized, and densitometric quantification of immunoreactive bands was performed using UN-SCAN-IT gel (version 4.3, Silk Scientific). Photoshop software was utilized to construct the figure from the raw western blot data.

### Immunohistochemistry

Rats were anesthetized at various time points after gene transfer with 50mg/kg of pentobarbital sodium (i.p) and perfused transcardially with 0.9% saline solution followed by 4% paraformaldehyde in 0.1M phosphate buffer solution. The brainstem and cervical spinal cord (C1-C2) was removed and immersed in the same fixative overnight at 4°C and transferred to 30% sucrose (w/v) in 0.1 M phosphate buffer solution for cryoprotection. After several days, 30 μm thick coronal sections of the tissue sample were sectioned with a cryostat at −20°C. Free floating tissue sections of the Vi/Vc were incubated with rabbit anti-5-HT (1:4000, Immunostar, Hudson,WI) antibody overnight at room temperature. After serial washes, the sections were incubated with Cy2-conjugated goat anti-rabbit IgG (1:500, Jackson ImmunoResearch, West Grove, PA). Control staining procedure was performed by omission of the primary antibody. Photoshop software was utilized to construct the figure from the original microscope photos.

### Vc lesion

Vc lesions were made using 1 μg/0.5 μl of ibotenic acid injected into the Vc unilaterally [14]. Rats were anesthetized and a 1-μl Hamilton syringe was placed between C1-C2 vertebrae and ibotenic acid was injected into the ipsilateral Vc over a 2-min period. The rats were allowed to heal over 5–7 days. CFA was injected into the ipsilateral side and behavioral testing was performed after 24 h. Ibotenic acid was reconstituted using deionized water.

### CFA and behavioral testing

Behavioral tests were conducted under blind conditions as described elsewhere [52]. A series of calibrated von Frey filaments were applied to the skin above the masseter muscle. Filaments were applied to the masseter muscle in increasing forces. Each von Frey filament was applied 5 times at intervals of a few seconds. A positive response was regarded as an active withdrawal of the head from the probing filament. The response frequencies [(number of responses/number of stimuli) X100%] to a range of von Frey filament forces were determined and a stimulus–response (S-R) curve plotted. After a non-linear regression analysis (GraphPad Prism), an EF_50_ value, defined as the von Frey filament force (g) that produces a 50% response frequency, was derived from the S-R curve. We used EF_50_ values as a measure of mechanical sensitivity. A leftward shift of the S-R curve, resulting in a reduction of EF_50_, occurred after inflammation [14]. This shift of the curve suggests the presence of mechanical hyperalgesia and allodynia since there was an increase in response to suprathreshold stimuli and a decreased response threshold for nocifensive behavior. Data are presented as mean ± S.E.M. Statistical comparisons were made by two-way ANOVA and ANOVA with repeated measures and post hoc comparisons (Neuman-Keuls). P<;0.05 was considered significant.

## Abbreviations

TMD: Temporomandibular joint disorder; CFA: Complete Freund’s adjuvant; STN: Spinal trigeminal nucleus; Vc: Subnucleus caudalis of the STN; Vi/Vc: Subnucleus caudalis/subnucleus interpolaris transition zone of the spinal trigeminal nucleus; RVM: Rostral ventromedial medulla; EF_50_: The effective force that produces 50% response frequency; IL-1β: Interleukin-1β; IL-1R: Interleukin-1 receptor; IL-1ra: Interleukin-1 receptor antagonist; NK1-R: Neurokinin-1 receptor; Tph-2: Tryptophan hydroxylase-2; i.p: intraperitoneal.

## Competing interests

The authors declare that they have no competing interests.

## Authors’ contributions

BC was involved in carrying out the behavioral, pharmacological, RNAi, immunostaining, and histological studies. BC was also involved in the experimental design and drafted the manuscript. WG was involved in the experimental design and RNAi and Western blot studies. FW contributed to the experimental design and the RNAi and immunostaining experiments. KR and RD conceived the study, participated in its design and coordination, and helped to draft the manuscript. All authors read and approved the final manuscript.
